# Administration of protopine prevents mitophagy and acute lung injury in sepsis

**DOI:** 10.3389/fphar.2023.1104185

**Published:** 2023-06-08

**Authors:** Zhong Xiao, Juan Long, Jie Zhang, Zhimin Qiu, Chen Zhang, Hongbing Liu, Xinyong Liu, Kang Wang, Yahui Tang, Longwang Chen, Zhongqiu Lu, Guangju Zhao

**Affiliations:** ^1^ Emergency Department, The First Affiliated Hospital of Wenzhou Medical University, Wenzhou, China; ^2^ Wenzhou Key Laboratory of Emergency and Disaster Medicine, Wenzhou, China; ^3^ The Key Specialty of Traditional Chinese Medicine of Zhejiang Provincial in the 13th Five-Year Plan Period (Emergency Department), Wenzhou, China

**Keywords:** sepsis, acute lung injury/ALI, protopine/PTP, inflammation, mitophagy

## Abstract

**Introduction:** Sepsis is a severe life-threatening infection that induces a series of dysregulated physiologic responses and results in organ dysfunction. Acute lung injury (ALI), the primary cause of respiratory failure brought on by sepsis, does not have a specific therapy. Protopine (PTP) is an alkaloid with antiinflammatory and antioxidant properties. However, the function of PTP in septic ALI has not yet been documented. This work sought to investigate how PTP affected septic ALI and the mechanisms involved in septic lung damage, including inflammation, oxidative stress, apoptosis, and mitophagy.

**Methods:** Here, we established a mouse model induced by cecal ligation and puncture (CLP) and a BEAS-2B cell model exposed to lipopolysaccharide (LPS).

**Results:** PTP treatment significantly reduced mortality in CLP mice. PTP mitigated lung damage and reduced apoptosis. Western blot analysis showed that PTP dramatically reduced the expression of the apoptosis-associated protein (Cleaved Caspase-3, Cyto C) and increased Bcl-2/Bax. In addition, PTP decreased the production of inflammatory cytokines (IL-6, IL-1β, TNF-α), increased glutathione (GSH) levels and superoxide dismutase (SOD) activity, and decreased malondialdehyde (MDA) levels. Meanwhile, PTP significantly reduced the expression of mitophagy-related proteins (PINK1, Parkin, LC-II), and downregulated mitophagy by transmission electron microscopy. Additionally, the cells were consistent with animal experiments.

**Discussion:** PTP intervention reduced inflammatory responses, oxidative stress, and apoptosis, restored mitochondrial membrane potential, and downregulated mitophagy. The research shows that PTP prevents excessivemitophagy and ALI in sepsis, suggesting that PTP has a potential role in the therapy of sepsis.

## 1 Introduction

Sepsis is a clinical disease characterized by an imbalanced host response to infection, systemic inflammatory response, and dysfunction of numerous organ systems (Singer et al., 2016; Napolitano, 2018). In critically ill patients, acute lung injury (ALI), also known as acute respiratory distress syndrome (ARDS), is a major cause of morbidity and mortality. According to reports, sepsis is responsible for about 40% of ALI occurrence (Fowler et al., 2019). The etiology of ALI is multifactorial which involved inflammation, oxidative stress, apoptosis, and mitochondrial damage (Li et al., 2022; Xia et al., 2022; Li et al., 2023). Despite the use of many different treatment options, sepsis treatment remains a major challenge (Esposito et al., 2017; Liu et al., 2022).

As a comprehensive approach, traditional Chinese medicine (TCM) shows outstanding achievements in treating critical illness (Luo Y. et al., 2019). Sepsis, defined as a febrile illness, has been treated by a variety of traditional Chinese medicines. More and more studies have shown that TCM has prospects and potential in the treatment of sepsis. Several studies have examined the anti-inflammatory properties, improvements in microcirculation, protective properties, and immune function of Chinese herbal medicines (Fan et al., 2020). Therefore, we attempted to explore the natural compounds with potential therapeutic effect from TCM in the sepsis treatment.

Protopine (PTP), which is an isoquinoline alkaloid, is an effective compound of *Corydalis yanhusuo W. T. Wang* (Hiller et al., 1998). There are a number of pharmacological properties of PTP, such as anticancer properties, hepatoprotective properties, anti-inflammatory properties, and antioxidative effects (Saeed et al., 1997; Nie et al., 2021). As reported, PTP can reduce the production of pro-inflammatory chemokines by blocking the MAPK signaling pathway. Meanwhile, PTP alleviated carrageenan (CA)-induced paw edema in mouse models (Alam et al., 2019). Another investigation showed that PTP intervention decreased the production of inflammatory mediators in LPS-stimulated RAW264.7 cells (Bae et al., 2012). Additionally, PTP played a role in refractory prostate cancer by regulating mitochondria-mediated signaling pathways, including Bcl-2 family proteins (Chen et al., 2012). In addition to the research mentioned above, additional pertinent literature has provided reviews of the pharmacological properties of PTP (Huang et al., 2021). It is well known that inflammation and oxidative stress play an important role in the occurrence and development of sepsis. Although the pharmacological effects of PTP have been observed in numerous studies, it still remains unclear whether PTP could alleviate ALI in sepsis. Therefore, we speculate that the anti-inflammatory and antioxidant pharmacological properties of PTP may play a role in the treatment of ALI in sepsis.

Mitochondria are important participants in the life of eukaryotic cells, which are an indispensable energy generator for maintaining cell homeostasis. In addition to the metabolic functions of mitochondrial such as energy conversion, oxidative phosphorylation, and tricarboxylic acid cycle, mitochondria also damage cells. When mitochondria are damaged, high levels of Ca2+ and cytochrome c as well as mtDNA are released from mitochondria into the cytoplasm, which induces inflammation and initiates apoptosis (Ramzan et al., 2020; Mohsin et al., 2021). Therefore, sustaining cellular homeostasis and survival depends on ensuring mitochondrial quality control, such as appropriate removal of damaged mitochondria. An essential mechanism for mitochondrial quality control, mitophagy eliminates and degrades defective mitochondria to maintain cellular homeostasis (Onishi et al., 2021). Excessive mitophagy, however, may worsen mitochondrial dysfunction and result in cell death.

Numerous studies have shown that sepsis-related alterations in mitochondrial structure and function may be a significant factor in organ failure caused by sepsis (Arulkumaran et al., 2016). For instance, in lung cells from a mouse model of pneumonia, mitophagy encourages the removal of damaged mitochondria, promoting cell survival and having a therapeutic impact in sepsis (Suliman et al., 2017). However, mitophagy may also play a distinct role during sepsis and worsen sepsis outcomes (Lee et al., 2014; Ryter and Choi, 2015). Mitophagy regulation may provide a promising therapeutic strategy for pulmonary sepsis treatment as more evidence accumulates.

Therefore, the purpose of the current investigation was outlined to explore whether PTP has any preventive effects against septic ALI. In this article, we explore the potential connections between PTP, mitophagy, and lung injury during sepsis.

## 2 Materials and methods

### 2.1 Animals and group

All animal experimental procedures were carried out in compliance with the Animal Care and Use Committee as well as Ethics Committee of the First Affiliated Hospital of Wenzhou Medical University (Wenzhou, China). C57BL/6N mice (Male, 20–25 g, 6–8-week-old) were bought from the Animal Center of the Chinese Academy of Science (Shanghai, China). The mice were raised in cages with light and dark cycles, food, and tap water, at room temperature (20°C–22°C) and 50% humidity.

All mice were randomly divided into 4 groups as follows: sham group, sham + PTP group, CLP group, and CLP + PTP group. ALI model of sepsis was reproduced by operation of cecal ligation and puncture (CLP). Mice in sham and sham + PTP groups went through the cecum exposure excluding ligation and puncture. PTP, purchased from MedChemExpress (catalog NO. HY-N0793, CAS NO. 130-86-9, purity of 99.64%) was prepared due to the manufacturer’s protocol and administered orally to sham + PTP and CLP + PTP groups at 20 mg/kg 60 min before surgery. The mice of the four groups were anesthetized using isoflurane as follows: anesthesia induction concentration 3.0%–5.0%, maintenance concentration 2.0%–3.0%.

All of the mice in the four groups were slaughtered 24 h after the animal model was established, and following perfusion, the lung tissues were separated from the animals. The rats were euthanized as follows: inhaled 5% isoflurane, and mice were killed by rapid cervical dislocation when limb stimulation did not respond. Histological examination and transmission electron microscopy (TEM) scans were performed on each group of lung sections. Eyeball blood obtained from mice was centrifuged at 3,000 rpm for 20 min, then the serum was collected and refrigerated at −80°C. The right lung was lavaged three times with 200 µL of cold PBS through the trachea, with a recovery rate of 80%, in order to collect bronchoalveolar lavage fluid (BALF), which was used to determine the total protein content and cell count in the fluid.

### 2.2 Cell culture

BEAS-2B cells were cultured in Dulbecco’s Modified Eagle Medium (DMEM) supplemented with 10% fetal bovine serum (FBS), 1% antibiotic (streptomycin and penicillin), and then maintained in 5% CO2 at 37°C. After reaching 70%–80% confluence, cells were washed with phosphate-buffered saline (Gibco, Invitrogen, USA). Cells were treated with LPS (100 μg/mL, *E. coli* 055: B6, Solarbio Science & Technology, China), PTP (2.5 µM, HY-N0793, MCE, United States), or the equal volume of PBS before the media was changed to serum-free medium. After 24 h, cells were collected for research and analysis. Four experimental groups were created using the cells: the CTRL group, the PTP group, the LPS group, and the LPS + PTP group.

### 2.3 Hematoxylin–eosin (H&E) staining

H&E staining was used to determine the morphology of the lung samples taken from each group. The lung tissues were, in a nutshell, fixed with 10% formalin for 24 h, embedded in paraffin, sectioned to 5-µm-thick, dewaxed with xylene, and dehydrated in ethanol at graded concentrations. Slices were examined under a microscope (Nikon, Japan) after being stained with hematoxylin and eosin dye (Solarbio Science and Technology, China). A semi-quantitative scoring method was used to rate the severity of lung injury, and the lung injury score was computed as previously explained (Zhuang et al., 2021).

### 2.4 Lung wet-to-dry (W/D) ratio

Lung tissue was separated, immediately weighed (wet weight), and then baked for 48 h at 70°C until weight stabilized (dry weight). Finally, the wet weight was divided by the dry weight to determine the wet/dry ratio (W/D).

### 2.5 Oxidative stress assay

The levels of Glutathione (GSH, Solarbio Science & Technology, China) and malondialdehyde (MDA, Beyotime Biotechnology, China) and activity of superoxide dismutase (SOD, Beyotime Biotechnology, China) in mice lung tissues were measured owing to the manufacturer’s instruction.

### 2.6 Real-time quantitative reverse transcription polymerase chain reaction (qRT-PCR)

RNA from lung tissue was extracted using RNAsimple Total RNA Kit (Tiangen Biotech, China). The RevertAid First Strand cDNA Synthesis Kit (Thermoscientific, USA) was used for reverse transcription to synthesize the cDNA. The conditions of reverse transcription were 5 min at 25°C, 60 min at 42°C, and a final step of 5 min at 70°C. Finally, SYBR Green Supermix (BIO-RAD, USA)-based PCR detection equipment was used to conduct qRT-PCR. Results were statistically analyzed using 2^−ΔΔCT^ method, with gene expression level normalized for mRNA expression of GAPDH. All primer sequences are shown in Table 1.

**TABLE 1:** Sequences of real-time PCR primer.

Gene	Forward primer (5′–3′)	Reverse primer (5′–3′)
GADPH	AGG​TCG​GTG​TGA​ACG​GAT​TTG	GGG​GTC​GTT​GAT​GGC​AAC​A
IL-1β	GCT​GCT​TCC​AAA​CCT​TTG​AC	CTT​CTC​CAC​AGC​CAC​AAT​GA
IL-6	AGT​TGC​CTT​CTT​GGG​ACT​GA	CCT​CCG​ACT​TGT​GAA​GTG​GT
TNF-α	GTG​GGT​GAG​GAG​CAC​GTA​GT	CGA​TCA​CCC​CGA​AGT​TCA​GTA​G

### 2.7 Enzyme-linked immunosorbent assay (ELISA)

Based on the manufacturer’s instructions, a standardized ELISA kit (Boyun, China) was used to quantify the levels of IL-6, IL-1βand TNF-α in serum and supernatant. Briefly, samples were put to an ELISA plate and then incubated for 30 min at 37°C with biotinylated antibody, followed by avidin-HRP. After washing and staining, the absorbance at 450 nm of each well was measured.

### 2.8 TUNEL assay

Apoptotic cells were examined by terminal deoxynucleotidyl transferase-mediated dNTP nick-end labeling (TUNEL). Following the manufacturer’s instructions for an *In Situ* Cell Death Detection kit, the TUNEL approach was used (Roche, USA). DAPI was used to label the nuclei. The ratio of apoptotic cells to total cells was used to represent the results.

### 2.9 Immunohistochemistry (IHC) analysis

Immunohistochemistry was used to analyze the expression of PINK1 in lung tissue. The sections underwent xylene deparaffinization, rehydration in graded alcohol solutions, and heat pretreatment for antigen repair. After quenching endogenous peroxidase and blocking non-specific proteins, sections were exposed to PINK1 antibody for an overnight incubation at 4°C. Finally, the sections were treated with a secondary antibody that was conjugated to HRP and stained with 3,3N-Diaminobenzidine Tertrahydrochloride (DAB, Beyotime Biotechnology, China). The images were observed at ×200 magnification under a light microscope.

### 2.10 Cell counting Kit-8 (CCK-8) assay

For measurement of cell viability, BEAS-2B cells (5,000 cells/well) were sown in 96-well plates and cultured at 37°C with 5% CO_2_. After 24 h, cells were incubated for 24 h in serum-free medium with LPS and PTP. The manufacturer’s instructions were followed when 10 µL of CCK-8 solution (Dojindo Laboratories, Japan) was added. A microplate reader was then used to measure the absorbance at 450 nm (SpectraMax iD3, United States).

### 2.11 Reactive oxygen species (ROS) assay

Cells were incubated at 37°C 20 min with 10 μM fluorescence probe DCFH-DA (Beyotime Biotechnology, China), and then the fluorescence intensity was measured by use of immunofluorescence and flow cytometry.

### 2.12 Flow cytometry

The BEAS-2B cells were collected, washed with cold PBS, and then mixed in 195 µL Annexin V-FITC binding buffer containing 5 µL Annexin V-FITC and 10 µL PI for 20 min at room temperature according to the manufacturer’s instructions (Beyotime Biotechnology, China). Cell apoptosis was studied using flow cytometer (BD FACSCanto II, USA) and data were processed in the FlowJo software. Flow cytometry was used to measure the intracellular Mitochondrial membrane potential (MMP) following JC-1(Beyotime Biotechnology, China) labeling of the fluorescent probe following the manufacturer’s instruction.

### 2.13 Transmission electron microscopy (TEM)

Lung tissue of mice was fixed for 4 h at 4°C with 2.5% glutaraldehyde, then for 2 h at 20°C with 1% osmium tetroxide. The tissues were then dehydrated through graded concentrations of ethanol and acetone, embedded into Epon812 to be sectioned with an ultramicrotome, and stained with uranyl acetate and lead nitrate. A transmission electron microscope was used to view the slices (HITACHI, Japan).

### 2.14 Western blotting analysis

Proteins extracted from lung tissue and cell lysate were fractionated on SDS-PAGE (Solarbio Science & Technology, China) and subsequently transferred to 0.45-μm polyvinylidene fluoride (PVDF, Merck, United States) membranes, blocked at room temperature for 30 min with Protein Free Rapid Blocking Buffer, followed by incubation with primary antibodies at 4°C overnight, and then with appropriate peroxide-conjugated secondary antibodies for 1 h at room temperature. Finally, chemiluminescence reagent was employed (Thermo Fisher Scientific, USA) and bands were detected with a chemiluminescence imaging system (Thermo Fisher Scientific, United States). The density of each band was calculated using ImageJ software (National Institutes of Health, United States). Antibodies against Bcl-2 (1:1000, Abcam, United Kingdom), Bax (1:1000, Abcam, United Kingdom), Caspase-3 (1:1000, Cell Signaling Technology, United States), Cleaved Caspase-3 (1:1000, Cell Signaling Technology, United States), Cytochrome C (Cyto C, 1:1000, Abcam, United Kingdom), PINK1 (1:1000, Abcam, United Kingdom), Parkin (1:1000, Cell Signaling Technology, United States), LC3A/B (1; 1000, Cell Signaling Technology, United States), Mfn2 (1:1000, Abcam, United Kingdom), Drp1(1:1000; Cell Signaling Technology, United States), β-actin (1:1000; Cell Signaling Technology, United States), and GAPDH (1:5,000, Proteintech, China) were used.

### 2.15 Statistical analyses

All results are described as means ± standard deviation (S.D). Use one-way analysis of variance (one-way ANOVA) for multiple comparisons. Statistical analyses were carried out by GraphPad Prism 8.0 (GraphPad Software, United States). *p*-value < 0.05 indicated statistically significant.

## 3 Results

### 3.1 PTP improves survival and reduces lung damage in CLP mice

**FIGURE 1:**
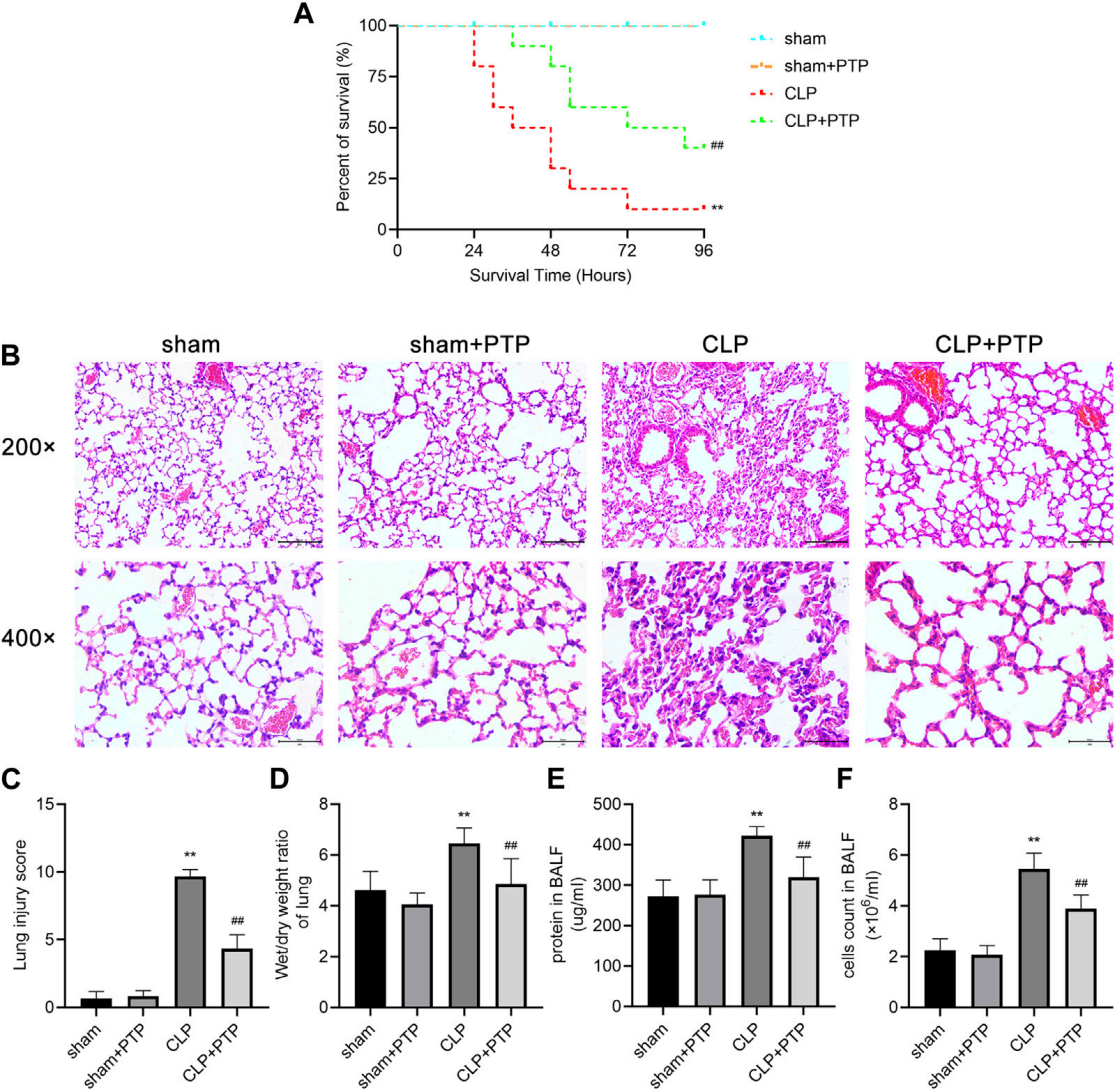
Effects of PTP on the survival and lung damage in CLP mice. **(A)** A study of the experimental mice’s survival was conducted (*n* = 10 per group; Kaplan–Meier survival analysis). **(B)** Representative H&E-stained sections under light microscope of 200 and 400 magnifications were shown to assess the injury severity of lungs. Scale bars = 100 µm. **(C)** Lung injury scores of H&E-stained lung tissue sections. **(D)** W/D ratios in mouse lung samples. **(E)** The protein content and **(F)** total cell numbers of BALF were measured. Data are shown as mean ± S.D (n ≥ 3 per group). ^*^ *p* < 0.05, ^**^ *p* < 0.01 vs. the sham group; ^#^ *p* < 0.05, ^##^ *p* < 0.01 vs. the CLP group.

Survival analysis was carried out to verify the protective effect of PTP in CLP mice. The CLP group had a 96-h survival rate of 10% (1/10), but the CLP + PTP group had a survival rate of 40% (4/10). In CLP mice, PTP therapy drastically reduced mortality (Figure 1A). The findings of H&E staining revealed histological features and associated alterations. The principal characteristics of lung injury, which were evident in the lung tissue of septic mice, were parenchyma edema, alveolar hemorrhage, massive infiltration of inflammatory cells into the interstitium, destruction of alveolar structures, and lung injury severity was then assessed using histopathological scores. The pathological damage was less severe in the CLP + PTP group than in the CLP group following PTP therapy. With lower pathological scores in the CLP + PTP group compared to the CLP group, the lung injury scores represent a similar level of lung injury (Figures 1B, C).

In order to calculate the vascular permeability index for each group, as shown in Figure 1D, we examined the W/D ratios. Mice in the CLP group had a much higher W/D ratio than those in the CLP + PTP group, whereas the W/D ratio was significantly reduced by PTP therapy (Figure 1D). Figures 1E, F shows a clear rise in the protein content and overall cell count in the BALF of the CLP group. However, PTP-treated reversed the levels of protein and total cells in BALF (Figures 1E, F).

### 3.2 PTP lowers apoptosis in the lungs of septic mice

**FIGURE 2:**
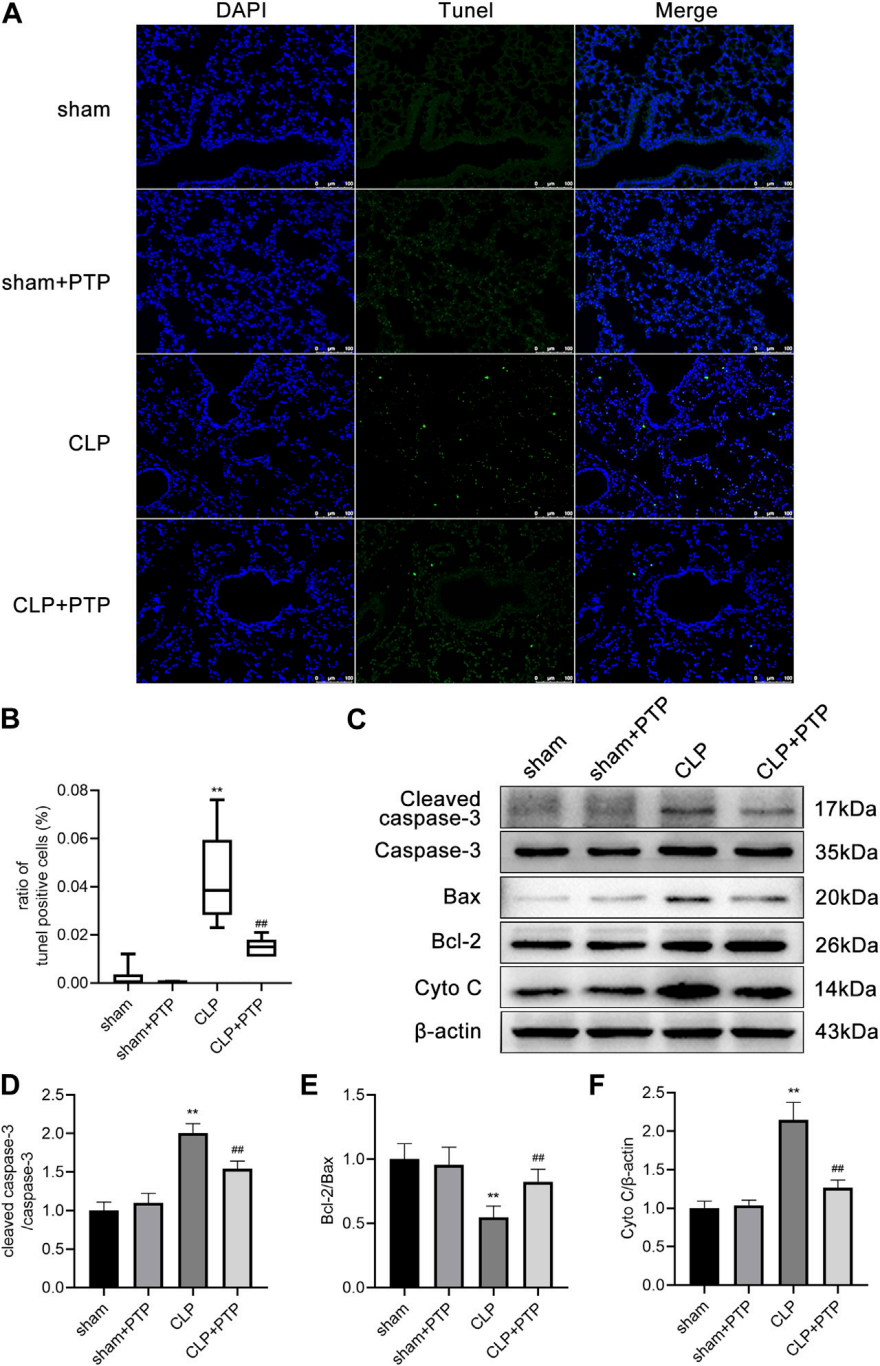
Effects of PTP on the regulation of apoptosis in the lung tissue of CLP mice. **(A)** Representative TUNEL-stained pictures are shown to evaluate the level of apoptosis in the lungs for each group. Scale bars = 100 µm. **(B)** Analysis of the ratio of TUNEL-positive cells. **(C)** Protein expression levels of Cleaved caspase-3, Caspase-3, Bax, Bcl-2, and Cyto C were determined by western blot. **(D–F)** Quantitative analysis of Cleaved caspase-3/Caspase-3, Bcl-2/Bax, and Cyto **(C)**. Data are shown as mean ± S.D (n ≥ 3 per group). ^*^ *p* < 0.05, ^**^ *p* < 0.01 vs. the sham group; ^#^ *p* < 0.05, ^##^ *p* < 0.01 vs. the CLP group.

We used TUNEL staining to observe lung tissue apoptosis in each group of mice to determine whether PTP has an inhibitory effect on the lung cell apoptosis in septic lung injury. After the CLP induction, the fraction of TUNEL-positive cells was increased significantly in mice lung tissue, but PTP treatment greatly reduced the proportion of apoptotic cells in mouse lung tissue (Figures 2A, B).

Then, using western blotting, we observed the protein expression of Bax, Bcl-2, Caspase-3, Cleaved Caspase-3, and Cyto C in the lungs. Bcl-2/Bax ratio was significantly lower in the CLP surgery group than it was in the sham operation group. Compared to lung tissue from the sham surgery, the expression of cleaved caspase-3/caspase-3 and cytochrome C was significantly higher in the CLP group. The apoptosis-related proteins in the CLP + PTP group dramatically increased following PTP intervention. (Figures 2C–F).

### 3.3 PTP attenuates inflammation and oxidative stress in the lung tissue of septic mice

**FIGURE 3:**
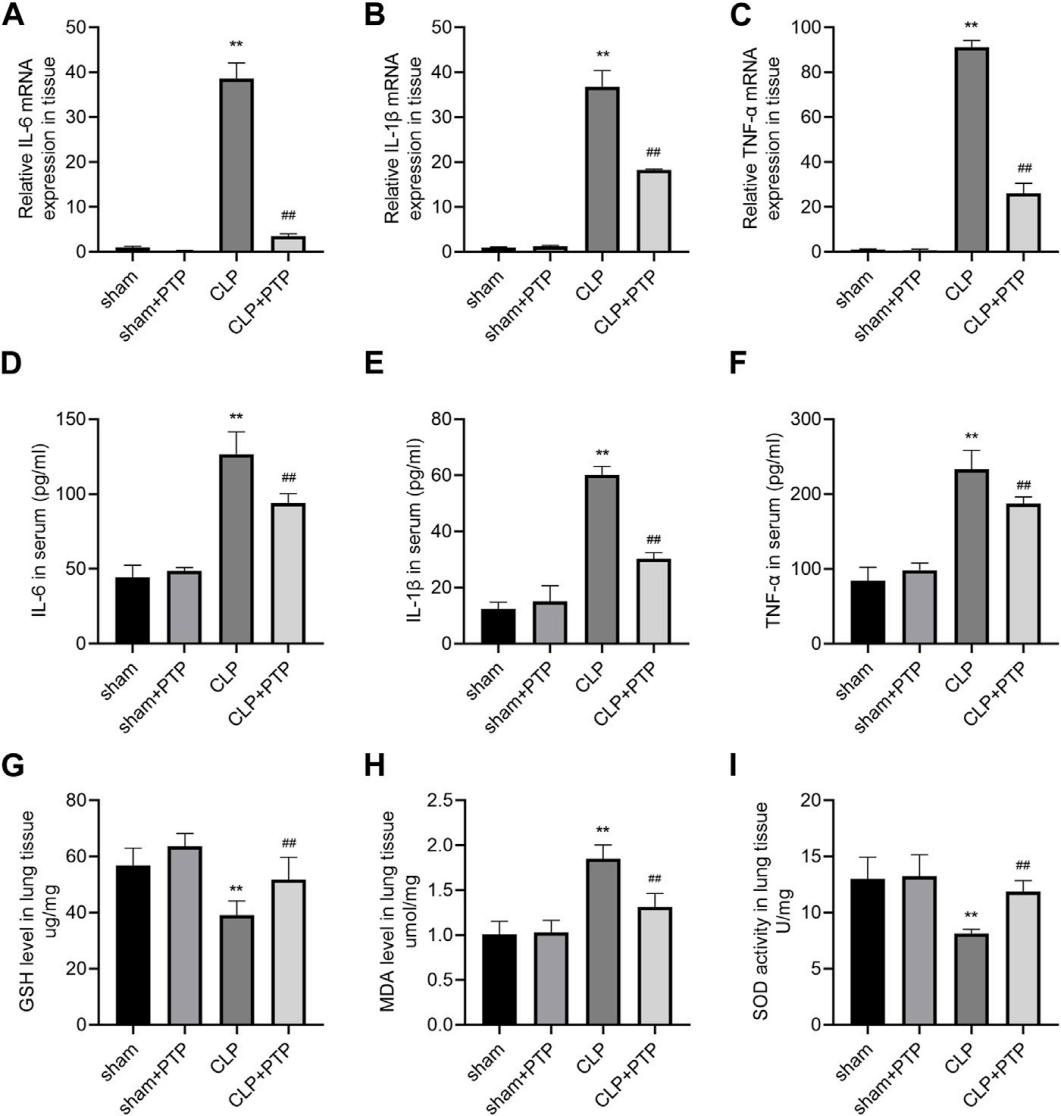
Effects of PTP on inflammatory response and oxidative stress in CLP mice. **(A–C)** The relative mRNA expression levels for IL-6, IL-1β, and TNF-α were examined in lung tissue by qRT-PCR. **(D–F)** Concentrations of IL-6, IL-1β, and TNF-α were measured in sera by ELISA. The levels of **(G)** GSH and **(H)** MDA and the activity of **(I)** SOD in lung tissue were measured. Data are shown as mean ± S.D (n ≥ 3 per group). ^*^ *p* < 0.05, ^**^ *p* < 0.01 vs. the sham group; ^#^ *p* < 0.05, ^##^ *p* < 0.01 vs. the CLP group.

Massive inflammatory factor release is intimately related to the emergence of septic lung damage. qRT-PCR and ELISA were used to determine the degree of pro-inflammatory cytokine expression in the lung tissues and serum, namely, IL-6, IL-1β, and TNF-α. As shown in Figures 3A–C, the relative mRNA expression levels of the cytokines were considerably elevated in the CLP group and significantly decreased following PTP therapy (Figures 3A–C). The CLP-induced mice had considerably higher serum concentrations of these pro-inflammatory factors, while animals treated with PTP had significantly lower serum levels of IL-6, IL-1β, and TNF-α (Figures 3D–F).

CLP significantly changes cellular oxidative stress, and excessive depletion of GSH in cells leads to mitochondrial oxidative stress and dysfunction. The most significant sulfhydryl antioxidant in cells, GSH, is essential for transporting amino acids, protecting proteins from sulfhydryl damage, and acting as an antioxidant. The level of GSH was significantly reduced in CLP-induced mice lung tissue (Figure 3G). MDA level was markedly increased in the lung of CLP mice (Figure 3H). Significantly less SOD, a vital antioxidant, was present in the CLP group (Figure 3I). However, PTP therapy dramatically decreased GSH and MDA levels as well as SOD activity in the CLP + PTP group, suggesting that PTP may be able to lessen oxidative stress.

### 3.4 PTP downregulates the mitophagy in CLP mice

**FIGURE 4:**
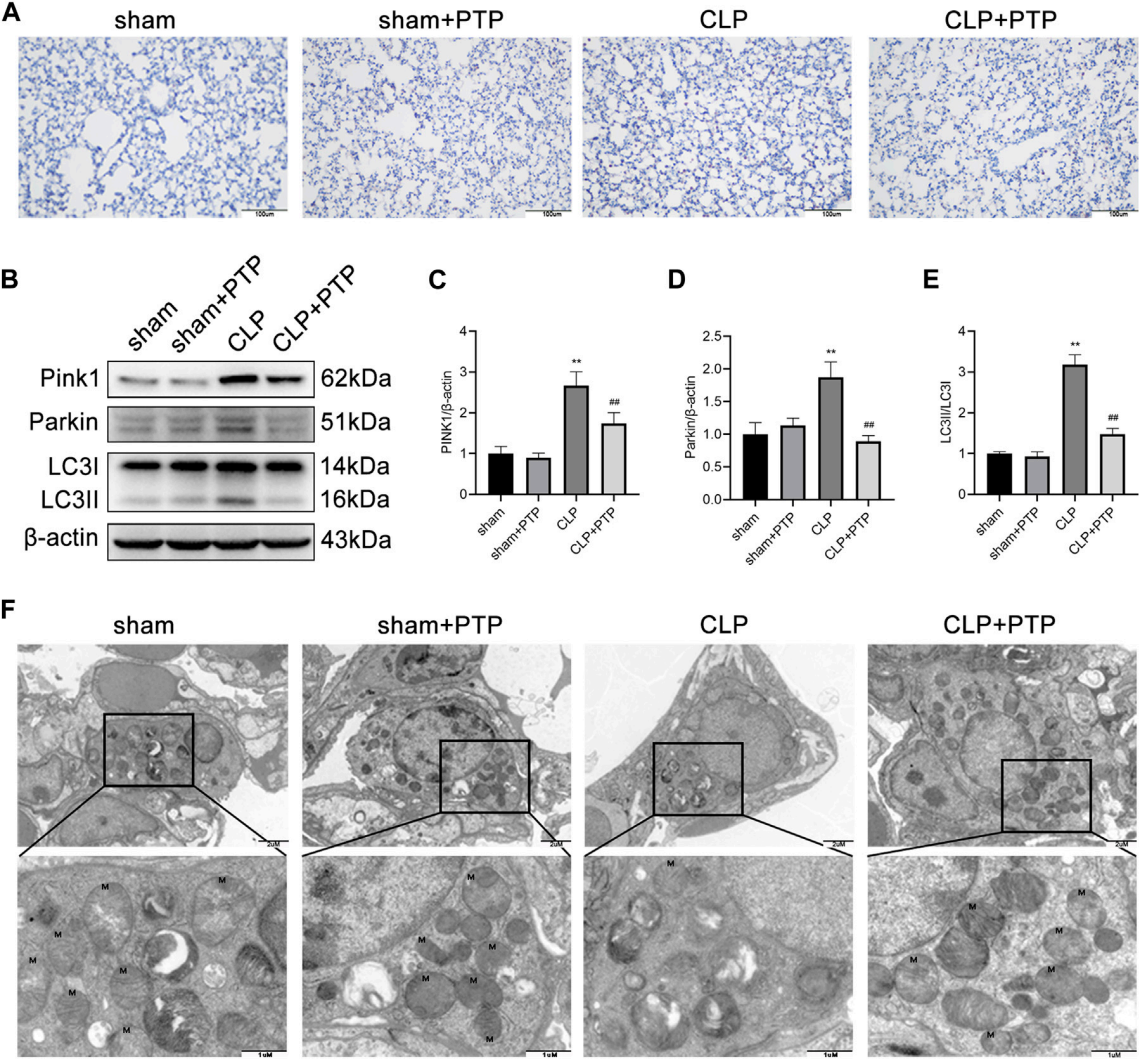
Effects of PTP on the mitophagy in CLP mice. **(A)** Immunohistochemistry staining of lung sections for PINK1. Scale bars = 100 µm. **(B)** The expression of PINK1, Parkin, LC3I, and LC3II was detected by western blotting. **(C–E)** Quantitative analysis of PINK1, Parkin, and LC3-II/LC3-I. **(F)** Transmission electron micrographs of mouse lung sections (×20,000, ×60,000). Data are shown as mean ± S.D (n ≥ 3 per group). ^*^ *p* < 0.05, ^**^ *p* < 0.01 vs. the sham group; ^#^ *p* < 0.05, ^##^ *p* < 0.01 vs. the CLP group.

To ascertain the mechanism by which PTP influences mitophagy activity, we focused on the expression of PINK1, an important protein for mitophagy. To evaluate the effect of PTP on the mitophagy, immunohistochemistry and western blotting were performed to determine the mitophagy-related proteins. As shown in Figures 4A–E, the expression of PINK1, total Parkin, and LC3-II/LC3-I were significantly increased after CLP-induced, whereas PTP treatment markedly reduced the expression of these proteins compared than in the CLP group (Figures 4A–E). These results indicated that an autophagic activation and mitophagy was involved in cell apoptosis of the septic ALI.

We then observed the ultrastructure of mitochondria under TEM. As shown in Figure 4F, the ultrastructure and number of mitochondria in the lung tissue of mice in the CLP group considerably altered in comparison to the sham group. After CLP-induced, the mitochondria of lung tissue cell were significantly swollen, and the cristae within the mitochondria were largely broken and reduced, and the number of mitochondrial was relatively small. After PTP treatment, the damage of mitochondrial was relieved, some cristae arrangement returned to regularity, fractures decreased, and the number of mitochondria increased (Figure 4F).

### 3.5 PTP ameliorate the cell apoptosis in LPS-stimulated BEAS-2B cells

**FIGURE 5:**
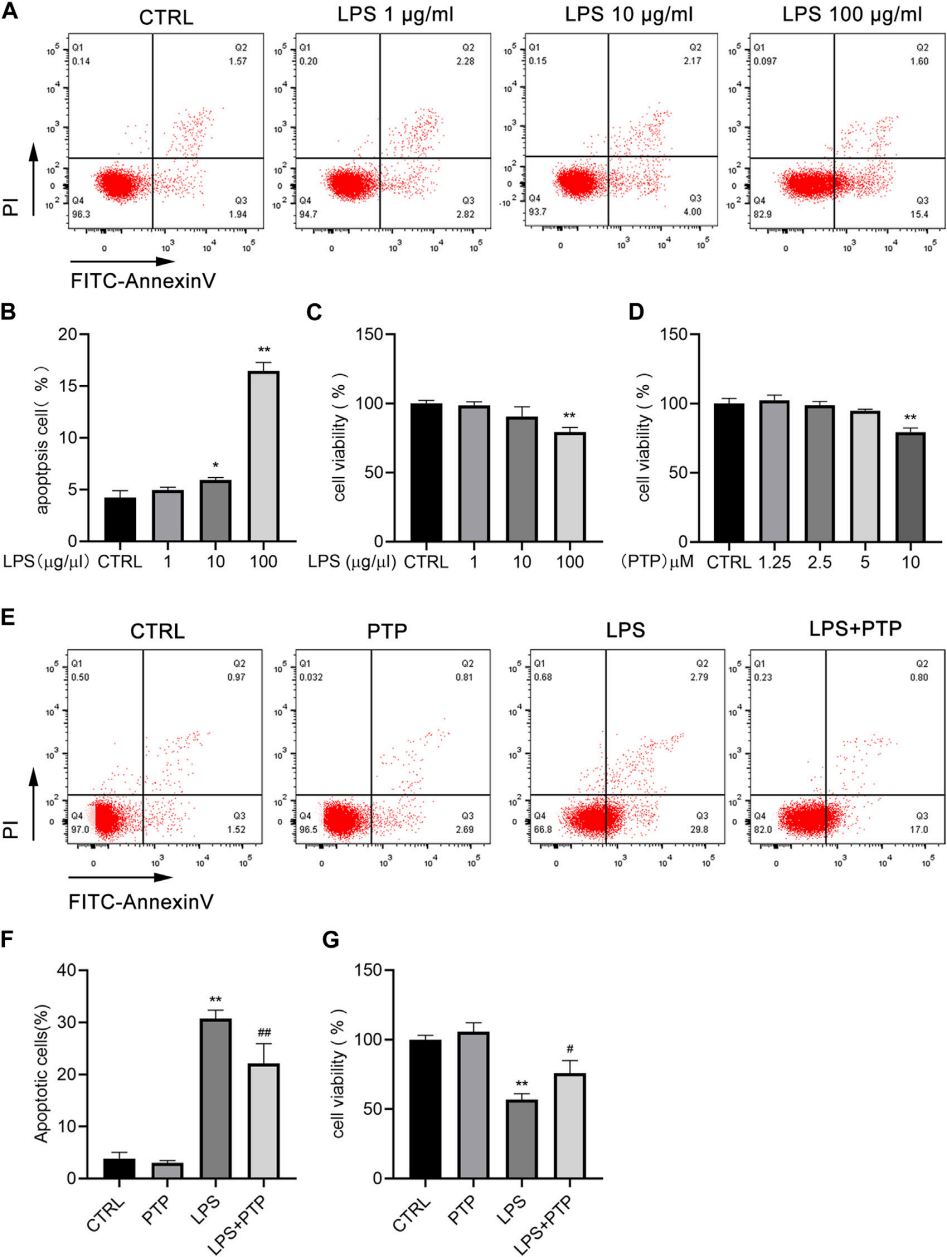
Effects of PTP on cell apoptosis in BEAS-2B cells induced by LPS. **(A)** Representative flow cytometry dot plot of apoptosis at various dosages of LPS. **(B)** Quantitative presentative of apoptosis at different concentrations of LPS. **(C)** BEAS-2B cell viability at different concentrations of LPS. **(D)** Effect of PTP on BEAS-2B cell viability. **(E)** Representative flow cytometry dot plot of changed cell apoptosis. **(F)** Quantitative presentative of apoptosis in each group. **(G)** Effect of PTP on BEAS-2B cell viability from each group. Data are shown as mean ± S.D (n ≥ 3 per group). ^*^ *p* < 0.05, ^**^ *p* < 0.01 vs. the control group; ^#^ *p* < 0.05, ^##^ *p* < 0.01 vs. the LPS group.

We treated cells with various concentrations of LPS at 1, 10, and 100 μg/mL for 24 h to examine the impact of LPS on the apoptosis of BEAS-2B cells. As shown in Figures 5A, B, the apoptosis of BEAS-2B cells was dose-dependent, with the increase of LPS concentration, the apoptosis also increased which was most obvious when the concentration reached 100 μg/mL (Figures 5A, B). Therefore, 100 μg/mL was selected as the experimental concentration. The results of the CCK-8 assay, which we performed to confirm the impact of LPS on the viability of BEAS-2B cells, were in line with what was previously stated (Figure 5C). Furthermore, to investigate the concentration ranges of PTP, we used CCK-8 assay in BEAS-2B cells. PTP was applied to the cells at a variety of dosages ranging from 1.25 to 10 µM for 24 h. As shown in Figure 5D, when treated with PTP up to 5 μM, there was no statistically significant effect on cell viability (Figure 5D). However, we used 2.5 µM as the experimental concentration. Then, we evaluated the effect of PTP on LPS-stimulated BEAS-2B cell injury using flow cytometry and CCK-8. The outcomes demonstrated that PTP dramatically decreased the apoptosis brought on by LPS and restored cell viability, both of which were consistent (Figures 5E–G).

### 3.6 PTP suppresses the inflammation and oxidative stress in BEAS-2B cells induced by LPS

**FIGURE 6:**
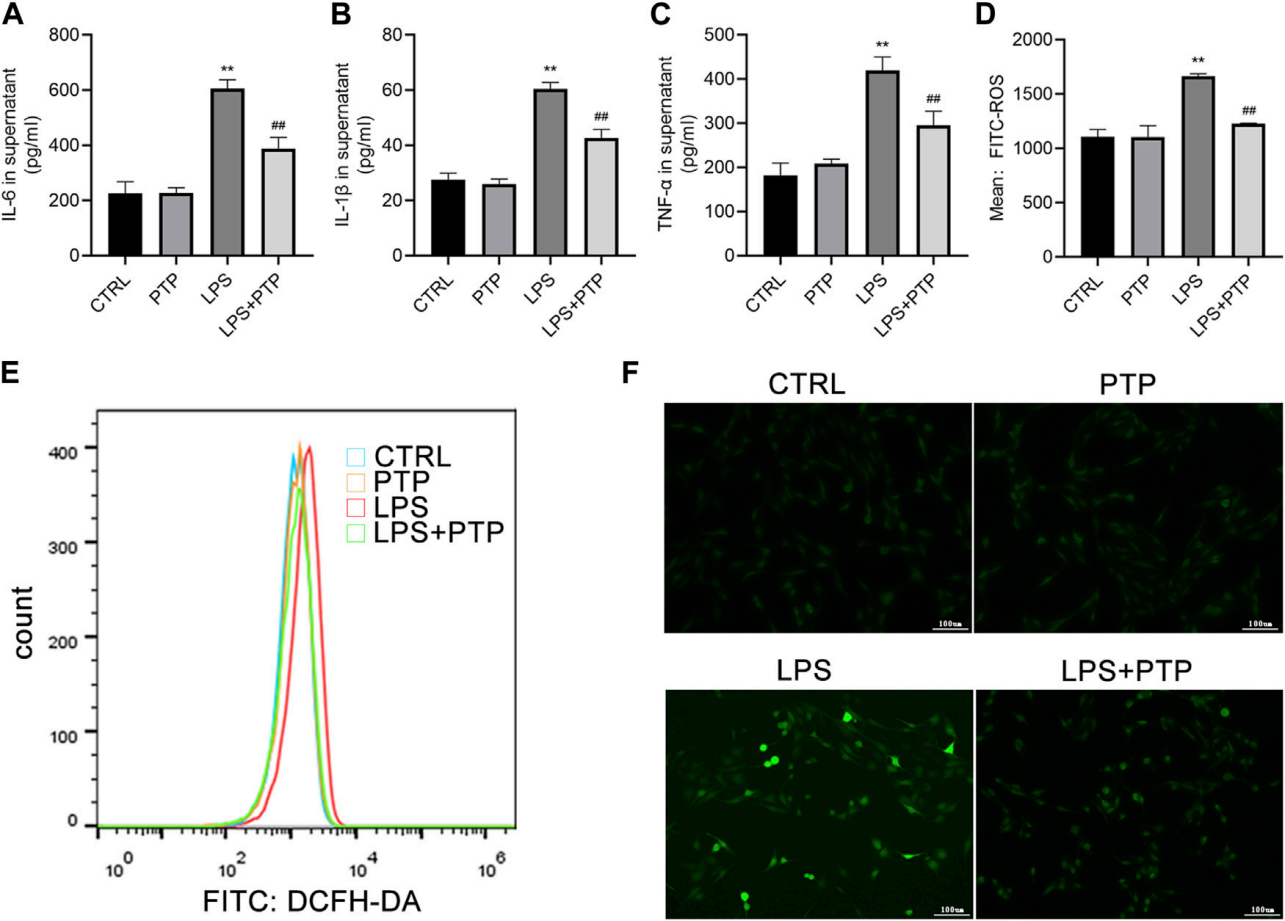
Effects of PTP on inflammatory response and oxidative stress to BEAS-2B cells. **(A–C)** Concentrations of IL-6, IL-1β, and TNF-α were measured in supernatant by ELISA. **(D, E)** The level of intracellular ROS was examined by flow cytometry. **(F)** The ROS level in BEAS-2B cells was examined by DCFH (×100). Data are shown as mean ± S.D (*n* ≥ 3 per group). ^*^ *p* < 0.05, ^**^ *p* < 0.01 vs. the control group; ^#^ *p* < 0.05, ^##^ *p* < 0.01 vs. the LPS group.

IL-6, IL-1β, and TNF-α levels of pro-inflammatory cytokine expression in the cell supernatant were assessed using ELISA. The expression levels of the cytokines were noticeably increased in the LPS group and drastically lowered after PTP treatment (Figures 6A–C). Then, alterations in the generation of intracellular ROS allowed the identification of the oxidative stress state in BEAS-2B cells. Results demonstrated that LPS treatment enhanced intracellular ROS generation whereas PTP intervention lowered it when combination with flow cytometry and immunofluorescence (Figures 6D–F).

### 3.7 PTP downregulates mitophagy in LPS-induced BEAS-2B cells

**FIGURE 7:**
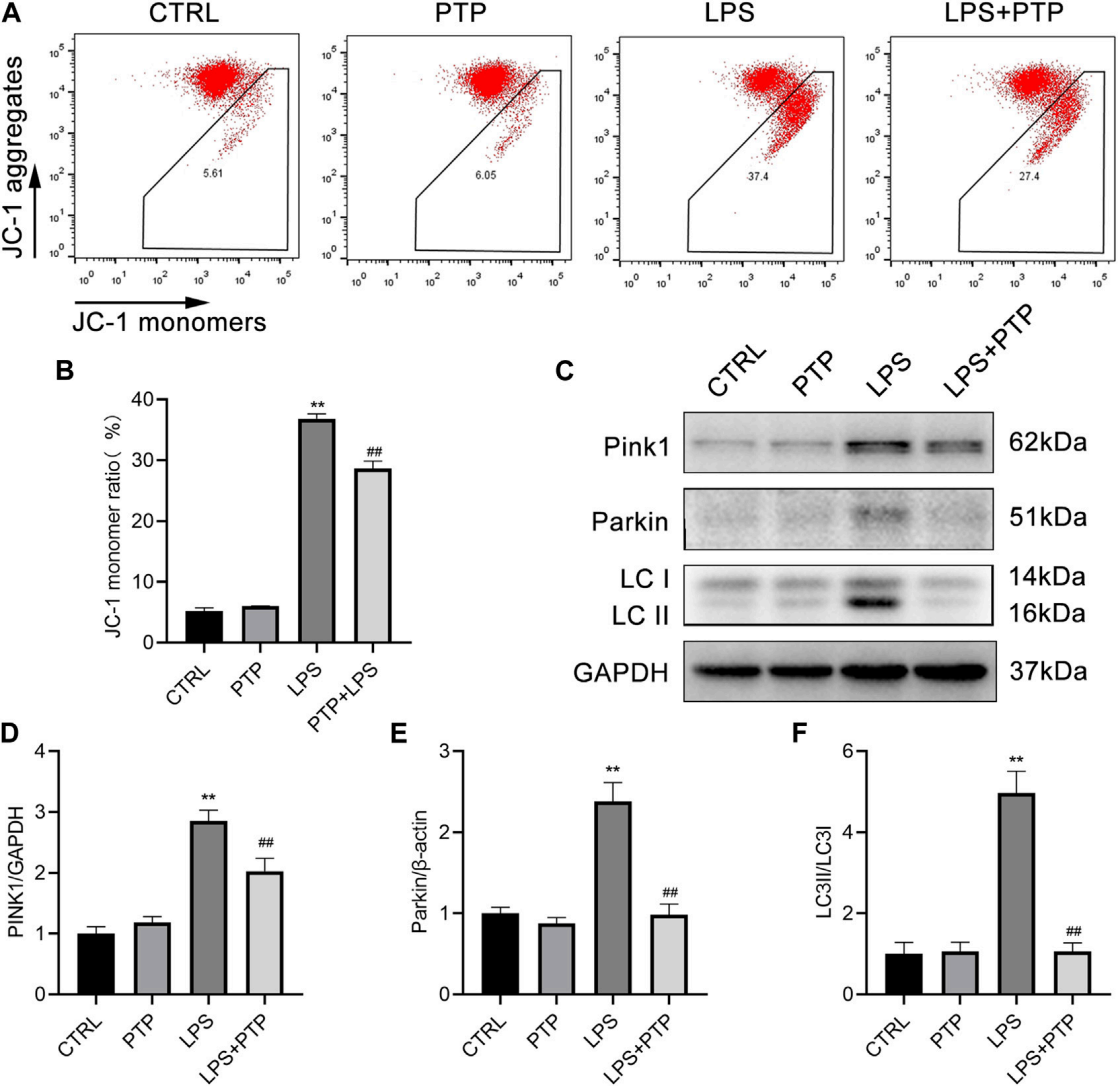
Effects of PTP on the mitophagy in BEAS-2B cells driven by LPS. **(A)** Representative flow cytometry dot plot of changed MMP by labeling with JC-1. **(B)** Quantitative presentation of JC-1 monomers ratio. **(C)** The protein expression levels of PINK1, Parkin, LC3I, and LC3II were detected by western blot. **(D–F)** Quantitative analysis of PINK1, Parkin, and LC3-II/LC3-I. Data are shown as mean ± S.D (n ≥ 3 per group). ^*^ *p* < 0.05, ^**^ *p* < 0.01 vs. the control group; ^#^ *p* < 0.05, ^##^ *p* < 0.01 vs. the LPS group.

As shown in Figure 7, PTP played important roles in alleviating the mitochondrial damage caused by LPS in BEAS-2B cells. Damage to the intracellular mitochondria was caused by LPS exposure, which significantly increased the proportion of JC-1 monomers in BEAS-2B cells compared to the control group. However, after PTP treatment, the proportion of intracellular JC-1 monomers was significantly reduced and the mitochondrial membrane potential (MMP) was reversed, indicating that the mitochondrial damage was mitigated (Figures 7A, B). In addition, we detected the expression of PINK1, total Parkin and LC3-II/LC3-I, which are major proteins for mitophagy+. As shown, after LPS induction, the expression of PINK1, Parkin and LC3-II/LC3-I increased more than the control group. Moreover, PTP treatment inhibited the expression of PINK1, Parkin and LC3-II/LC3-I in LPS-exposure cells (Figures 7C–F). The results were consistent with those in animal tissues.

## 4 Discussion

PTP, an alkaloid purified from traditional Chinese medication, was discovered to have pharmacological protective effects against inflammation and oxidative stress (Alam et al., 2019; Huang et al., 2021). According to Beibei Zhang et al., PTP can reduce the effects of acute kidney injury (AKI) brought on by the activation of Toll-like receptors 4 (TLR4) (Zhang et al., 2019). Another study revealed that PTP had effects on inhibition of the inflammatory activity of LPS-stimulated macrophages (Bae et al., 2012). PTP possesses anticancer properties as well. PTP reduced the activity of hepatocellular cancer cells in a caspase-dependent manner and induced apoptosis through an endogenous mechanism. Meanwhile, it prevented tumor development in mice with xenografts while exhibiting no overt toxicity (Nie et al., 2021). Another study demonstrated that the usage of PTP is safe by examining the effects of protopine on cytochrome P450 (CYPS) 1A1 and 1A2 mRNA levels, CYP1A protein, and activity levels in human hepatocytes in hepatoma HepG2 cells (Vrba et al., 2011). In this work, we discovered a unique function for PTP in ameliorating septic ALI. This protective effect of PTP was highly due to the correction of excessive mitophagy.

Lung is the most common organ failure in sepsis, and ALI is related to significant morbidity and mortality (Kumar, 2020). In this work, we created a septic ALI model in mice using CLP. CLP has grown to be the most popular rodent sepsis experimental model and is generally recognized as the gold standard for sepsis research, reflecting many characteristics of human sepsis (Rittirsch et al., 2009). Excessive inflammation in sepsis leads to the early onset of organ damage, and lung injury is significantly influenced by the presence of inflammatory cytokines (Vishnupriya et al., 2020). Lung dysfunction and cell damage are encouraged by cytokine-mediated inflammation, which contributes to the development of ALI (Goodman et al., 2003). The lung tissue showed impaired morphology, and the pathological score in CLP group was significantly increased, but PTP treatment alleviated the pathological injury. In this article, sepsis dramatically increased the levels of the inflammatory cytokines IL-1β, IL-6, and TNF-α, although this rise was reduced following PTP treatment. Besides the inflammatory factors, PTP treatment significantly reduced the W/D ratio, alleviated pulmonary edema in CLP mice, reduced total protein and cell infiltration in BALF, and decreased the vascular permeability index of lung tissue, which also indicated that PTP could alleviate the pulmonary inflammation in sepsis. But as a drawback of the experiment, we did not categorize the different types of inflammatory cells and look at the quantity of inflammatory factors in the BALF.

There is a correlation between oxidative stress and lung injury, and lung injury is accompanied by oxidative stress disorder (Galley, 2011). When mice are in sepsis, the lung is exposed to inflammatory condition, leading to cellular oxidative stress disorder, the production of massive ROS causes mitochondria dysfunction and cell death. We found that the level of intracellular oxidative stress in mice lung tissue was significantly increased after CLP-induced, which accompanied by an increase in cell apoptosis, and PTP treatment ameliorated both oxidative stress and cell apoptosis. These data suggest that PTP alleviated CLP-induced oxidative stress and inhibited cell apoptosis.

The “power stations” of eukaryotic cells, mitochondria provide the majority of the energy required by organisms (Arulkumaran et al., 2016). In addition to producing energy, mitochondria are crucial for cell metabolism, proliferation, differentiation, growth and death. Additionally, the condition of mitochondrial structure and function determine cell survival and death (Bastarache et al., 2006; Murphy and Hartley, 2018). Mitochondrial dysfunction, which is a key cellular event leading multiple organ dysfunction syndrome (MODS), has been increasingly regarded as playing important roles in sepsis (Supinski et al., 2020). Mitophagy performs crucial functions in mitochondrial quality control by eliminating the damaged mitochondria as part of the process to maintain mitochondrial and cell homeostasis (Killackey et al., 2020). But excessive mitophagy can be harmful (Zhang et al., 2020; Zhuang et al., 2021). In pulmonary sepsis, mitophagy helps cells survive by eliminating damaged mitochondria, but it can also aggravate lung damage by upsetting mitochondrial homeostasis and inducing cell death.

The etiology of ALI is complex, and the role of mitophagy in ALI may have two sides. While excessive or insufficient mitophagy may be harmful and worsen the development of ALI, moderate mitophagy may act as a preventative measure against it. According to what was discovered by Xu Luo et al., reduction of mitophagy reduced cell apoptosis, oxidative stress, and inflammation in rats, alleviating ALI (Luo X. et al., 2019). In this work, we discovered that excessive mitophagy led to higher levels of inflammation, oxidative stress, compromised mitochondrial integrity, and malfunction. When mitochondrial structure and function are out of whack, too much ROS is produced, which in turn causes mitophagy to worsen. As a result, in present study, excessive mitophagy exacerbated septic ALI without contributing to the maintenance of normal mitochondrial function or cell homeostasis.

During mitochondrial depolarization, the PINK1 protein builds up on damaged mitochondria, which is related to the health and integrity of mitochondria. By translocating to the mitochondrial outer membrane (MOM) and recruiting Parkin from the cytoplasm to the membrane, PINK1 triggers the start of mitophagy. As predicted, the findings revealed that the expression of PINK1 and total Parkin were obviously upregulated after CLP, indicating that mitophagy has been activated via the PINK1/Parkin signaling pathway and involved in septic lung injury in the mice. Mitophagy levels were reversed by PTP treatment, which blocked the PINK1/Parkin signaling pathway. Furthermore, we used TEM to identify the morphology and quantity of mitochondria in mice lung tissue in order to determine whether PTP has the effect of reducing the dysfunctional and structure of mitochondria caused by sepsis. At 24 h after sham or CLP procedures, the ultrastructure and number of mitochondria in the CLP group drastically altered, the integrity of mitochondrial structure was destroyed, some mitochondria were noticeably swollen, and the crests were clearly fractured. After PTP intervention, the damage of mitochondria was lessened, some cristae arrangement returned to regularity, fractures decreased, and the number of mitochondria increased. This result provides evidence that in CLP-induced lung injury, PTP may regulate mitophagy via the PINK1/Parkin signaling pathway. In addition, the TUNEL method and the expression of apoptosis-related proteins were also employed to identify apoptosis in mouse lung tissue. TUNEL staining and protein expression levels revealed that PTP-treated was able to downregulate the rise in apoptosis that CLP-induced in the mice lung tissue. The expression of cytochrome c increased overall as a result of CLP, and it reduced as a result of PTP intervention. In light of the aforementioned findings, we hypothesized that PTP might lessen cell apoptosis and ameliorate lung damage by preventing excessive mitophagy.

We generated the inflammatory cell model by treating cells with LPS in addition to the CLP-induced animal model. Human airway epithelial cells are connected to the development of ALI brought on by sepsis (Pugin et al., 2008; Cabrera-Benitez et al., 2012). Therefore, we selected the BEAS-2B cell line as a representative human airway epithelial cell to study ALI in sepsis. In order to examine biological processes including apoptosis, inflammation, oxidative stress, and mitophagy, a cell model made of BEAS-2B cells was employed. The lung inflammatory response to sepsis is mimicked by the LPS-stimulated human normal epithelial cell line, a validated model of cellular damage (Koyama et al., 2000). The findings were in line with animal studies and demonstrated that PTP intervention dramatically reduced the inflammatory response, the formation of ROS and JC-1 monomers, and downregulated LPS-induced excessive mitophagy to restore cell viability and decrease apoptosis.

The work has some limitations, including the fact that we didn't address the control of mitophagy with PINK1 pathway inhibitors or activators. The function of mitophagy in PTP-treated septic ALI must thus be clarified further in the future.

**FIGURE 8:**
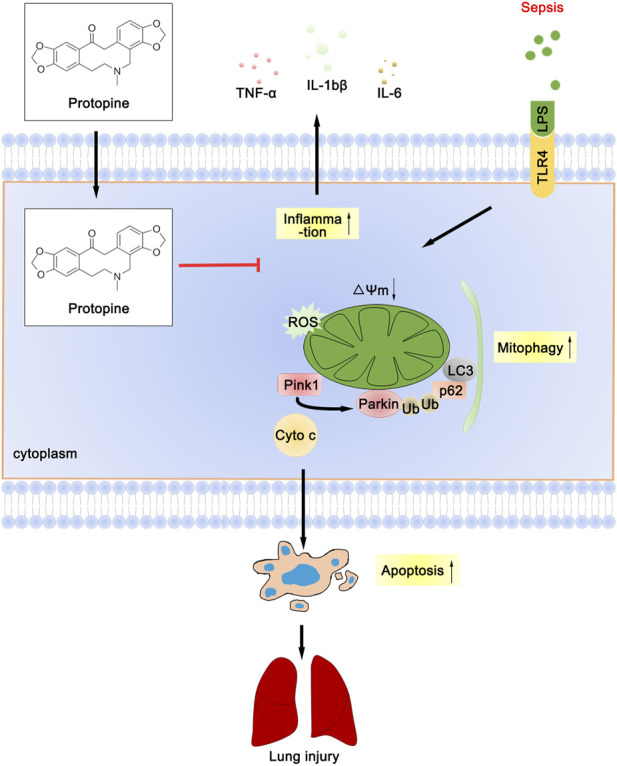
Schematic diagram depicts the molecular basis of PTP on ALI treatment. PTP can reduce apoptosis by regulating mitochondrial damage and alleviating mitophagy, and can also directly reduce apoptosis to treat septic lung injury.

Collectively, PTP reduced the inflammatory response of lung tissue, decreased the level of oxidative stress, downregulated the level of mitophagy, effectively prevented the septic ALI (Figure 8), which provides experimental and theoretical support for the use of PTP as a pulmonary sepsis therapy in the future.

## Data Availability

The original contributions presented in the study are included in the article/Supplementary Materials, further inquiries can be directed to the corresponding authors.
